# Investigation of the association between central arterial stiffness and aggregate *g*-ratio in cognitively unimpaired adults

**DOI:** 10.3389/fneur.2023.1170457

**Published:** 2023-04-24

**Authors:** John P. Laporte, Mary E. Faulkner, Zhaoyuan Gong, Elango Palchamy, Mohammad A.B.S. Akhonda, Mustapha Bouhrara

**Affiliations:** Laboratory of Clinical Investigation, National Institute on Aging, National Institutes of Health, Baltimore, MD, United States

**Keywords:** pulse wave velocity, myelin, arterial stiffness, quantitative MRI, aggregate g-ratio

## Abstract

Stiffness of the large arteries has been shown to impact cerebral white matter (WM) microstructure in both younger and older adults. However, no study has yet demonstrated an association between arterial stiffness and aggregate *g*-ratio, a specific magnetic resonance imaging (MRI) measure of axonal myelination that is highly correlated with neuronal signal conduction speed. In a cohort of 38 well-documented cognitively unimpaired adults spanning a wide age range, we investigated the association between central arterial stiffness, measured using pulse wave velocity (PWV), and aggregate *g*-ratio, measured using our recent advanced quantitative MRI methodology, in several cerebral WM structures. After adjusting for age, sex, smoking status, and systolic blood pressure, our results indicate that higher PWV values, that is, elevated arterial stiffness, were associated with lower aggregate *g*-ratio values, that is, lower microstructural integrity of WM. Compared to other brain regions, these associations were stronger and highly significant in the splenium of the corpus callosum and the internal capsules, which have been consistently documented as very sensitive to elevated arterial stiffness. Moreover, our detailed analysis indicates that these associations were mainly driven by differences in myelination, measured using myelin volume fraction, rather than axonal density, measured using axonal volume fraction. Our findings suggest that arterial stiffness is associated with myelin degeneration, and encourages further longitudinal studies in larger study cohorts. Controlling arterial stiffness may represent a therapeutic target in maintaining the health of WM tissue in cerebral normative aging.

## 1. Introduction

The stiffening of the large arteries contributes substantially to cardiovascular diseases in older individuals, while also being positively associated with various clinical symptoms and conditions including systolic hypertension, coronary artery disease, stroke, heart failure, and atrial fibrillation (1–4). Furthermore, arterial stiffness, commonly probed noninvasively with pulse wave velocity (PWV) (5, 6), has long been shown to be associated with a myriad of neurodegenerative diseases such as Alzheimer’s and dementias, and concomitant cognitive impairments (7–9). Indeed, mounting evidence indicates that age-related increases in the elastic stiffness of the arteries leads to abnormal changes in blood pulsatility, which can severely damage cerebral microcirculation and in turn result in reduced cerebral blood flow (CBF). Deficits in CBF have been linked to various neurodegenerative diseases (10), and have recently been shown to be associated with microstructural damages of cerebral tissues, including the myelin sheaths (11–13).

Magnetic resonance imaging (MRI) studies of normative aging and various neurological conditions have documented the relationship between arterial stiffness and cerebral structure, indicating an overall direct positive correlation between white matter lesions and central arterial stiffness (14–16). However, little attention has been paid to the relationship between arterial stiffness and cerebral microstructure. Yet, deteriorations in cerebral microstructure have been shown to precede the concomitant macrostructural changes (17). In seminal works using diffusion tensor imaging (DTI) (18, 19), an MRI technique sensitive to the underlying microarchitecture of the brain parenchyma and the degree and direction of water molecule mobility (20, 21), Turami and colleagues (22), Suri and colleagues (23), and Maillard and colleagues (24), have shown that, in both younger and older adults, elevated arterial stiffness, indicated by elevated PWV, was associated with lower values of fractional anisotropy (FA) and higher values of radial diffusivity (RD). FA reflects the directionality of molecular diffusion, where higher values are an indicator of anisotropic diffusion that has preferential direction, whereas RD reflects the magnitude of the molecular displacement by diffusion perpendicular to the axons, with higher values indicating more freely diffusing water (18, 19). These aforementioned alterations in cerebral microstructure with elevated arterial stiffness have been interpreted as deteriorations in axonal myelination.

However, despite being sensitive to cerebral microstructural changes, DTI indices, including FA and RD, are not specific. Indeed, multiple factors aside from myelination can affect the DTI-derived eigenvalues from which FA, RD and other DTI indices are calculated (25, 26); these include axonal degeneration, hydration, temperature, flow, macromolecular content and architectural features, including fiber fanning or crossing. A recent study by Badji and colleagues (27), found that higher values of PWV were shown to be associated with higher FA values and lower RD values, suggesting that, paradoxically, elevated arterial stiffness is associated with a higher level of cerebral microstructural tissue integrity. It remains unclear whether this intriguing discrepancy is due to study cohorts’ differences or methodological differences, requiring additional investigations. Moreover, using the magnetization transfer MRI measure, a sensitive but non-specific measure of myelin content, no significant association has been found between this MRI metric and PWV (27). Therefore, the direct association between arterial stiffness and myelination has not yet been established.

Building on these previous works, in this study, we examined the association between arterial stiffness, measured using PWV, and cerebral microstructure, probed using our recently introduced MRI method of aggregate *g*-ratio mapping (28). The *g*-ratio, defined as the inner-to-outer diameter of a myelinated axon, has been related to the speed of neural impulse conduction and provides information about the underlying myelination status of the axons (29, 30). This advanced MRI metric requires estimation of the myelin volume fraction (MVF) and axonal volume fraction (AVF), and has been shown to be a sensitive biomarker of cerebral microstructural tissue deteriorations in normative aging and various conditions, including Alzheimer’s disease and multiple sclerosis, with higher aggregate *g*-ratio values corresponding to greater deterioration of axonal myelination (28, 31–35). Additionally, in a secondary analysis, we investigated the association between PWV and MVF or AVF for a detailed examination of the dominant microstructural component underlying the differences seen in the association of aggregate *g*-ratio with PWV. Our investigation was conducted on a cohort of well-characterized cognitively unimpaired adults (*N* = 38), across the age range of 22 to 94 years.

## 2. Material and methods

### 2.1. Study cohort

Participants were drawn from the Baltimore Longitudinal Study of Aging (BLSA) and from the Genetic and Epigenetic Signatures of Translational Aging Laboratory Testing (GESTALT), two ongoing healthy aging cohorts at the National Institute on Aging (NIA). The study populations, experimental design, and measurement protocols of the BLSA have previously been reported. The BLSA is a longitudinal cohort study funded and conducted by the NIA Intramural Research Program (IRP). Established in 1958, the BLSA enrolls community-dwelling adults with no major chronic conditions or functional impairments. The GESTALT study is also a study of healthy volunteers, initiated in 2015 and funded and conducted by the NIA IRP. The goal of the BLSA and GESTALT studies is to evaluate multiple biomarkers related to aging. We note that the inclusion and exclusion criteria for these two studies are essentially identical. Participants underwent testing at the NIA’s clinical research unit and were excluded if they had metallic implants, neurologic, or medical disorders (36).

### 2.2. MR imaging

The calculation of the aggregate *g*-ratio requires two measures: the myelin volume fraction (MVF) and the axonal volume fraction (AVF), as described in detail previously (28, 37). Briefly, for the MVF mapping, we used the BMC-mcDESPOT protocol which consists of acquiring 3D spoiled gradient recalled echo (SPGR) images with flip angles (FAs) of [2 4 6 8 10 12 14 16 18 20]°, echo time (TE) of 1.37 ms, repetition time (TR) of ~5 ms, and acquisition time of ~5 min, as well as 3D balanced steady state free precession (bSSFP) images acquired with FAs of [2 4 7 11 16 24 32 40 50 60]°, TE of 2.8 ms, TR of 5.8 ms, and acquisition time of ~6 min. The bSSFP images were acquired with radiofrequency excitation pulse phase increments of 0 or *π* in order to account for off-resonance effects (38–42). All SPGR and bSSFP images were acquired with an acquisition matrix of 150 × 130 × 94, and voxel size of 1.6 mm × 1.6 mm × 1.6 mm. Further, we used the double-angle method (DAM) to correct for the excitation radio frequency inhomogeneity (43). For that, two fast spin-echo images were acquired with flip angles of 45° and 90°, echo time of 102 ms, repetition time of 3,000 ms, acquisition voxel size of 2.6 mm × 2.6 mm × 4 mm, and acquisition time of ~4 min. The acquisition time for the entire imaging protocol was ~21 min. For the AVF mapping, we used the NODDI protocol which consists of acquiring diffusion weighted images (DWI) with single shot EPI with TR of 10 s, TE of 67 ms, and three *b*-values of 0, 700, and 2,000 s/mm^2^, with the latter two encoded in 32 directions, and acquisition voxel size of 2 mm × 2 mm × 3 mm. The acquisition time was ~16 min. All images were obtained with field of view of 240 mm × 208 mm × 150 mm and reconstructed to a voxel size of 2 mm × 2 mm × 2 mm. The total acquisition time is ~37 min. We emphasize that all MRI studies and ancillary measurements were performed with the same MRI system, running the same pulse sequences, at the same facility, and directed by the same investigators for both BLSA and GESTALT participants. MRI scans were performed using a 3 T Philips MRI system (Achieva, Best, The Netherlands). Experimental procedures were performed in compliance with our local Institutional Review Board, and participants provided written informed consent.

### 2.3. Image segmentation and regions-of-interest determination

After thorough visual inspection of data quality for each participant, the scalp, ventricles, and other nonparenchymal regions within the images were eliminated using the BET tool as implemented in the FMRIB Software Library (FSL) (44). The averaged SPGR image over FAs for each participant was registered using nonlinear registration to the Montreal Neurological Institute (MNI) standard space image and the derived transformation matrix was then applied to the aggregate *g*-ratio, AVF and MVF maps for that corresponding participant using the FNIRT tool as implemented in FSL. Eleven regions of interest (ROIs) were defined from the MNI structural atlas. These ROIs included the three regions denoted from literature as being particularly vulnerable to increased central arterial stiffness, namely, the splenium of the corpus callosum (SCC), the internal capsule (IC) and the inferior longitudinal fasciculus (ILF) (27), as well as eight other main brain regions and tracts, namely, the whole brain (WB), frontal lobes (FL), occipital lobes (OL), parietal lobes (PL), temporal lobes (TL), anterior thalamic radiation (ATR), the corticospinal tract (CST) and the inferior fronto-occipital fasciculus (IFOF). All ROIs were eroded to reduce partial volume effects and imperfect image registration using the FSL tool *fslmaths*. Finally, for each ROI and each participant, mean aggregate *g*-ratio, AVF, and MVF values were calculated. Further details of our pipeline analysis can be found here (45–47).

### 2.4. Carotid-femoral pulse wave velocity

Using the SphygmoCor system (AtCor Medical, Sydney, Australia), carotid-femoral PWV was measured after the participants had rested in the supine position in a quiet room for at least 10 min (5). Subjects abstained from food or from drinking coffee or other caffeine-containing beverages for at least 45 min before PWV measurements. PWV was calculated as the distance traveled by the pulse wave divided by the time difference between the beginning of carotid and femoral arterial waveforms gated to electrocardiogram. The distance traveled by the pulse wave was measured to the nearest centimeter with an external tape measure over the body surface. This distance was measured by subtracting the distance between the manubrium and the carotid sampling site from the sum of the distances between the manubrium and the umbilicus and, between the umbilicus and the femoral sampling site. PWV measurements were repeated 3 times in each visit and the average was calculated (5). For each participant, the PWV was measured on the same day of the MRI scan.

### 2.5. Statistical analysis

For each ROI, the effect of PWV on aggregate *g*-ratio was investigated using a multiple linear regression model that incorporated the smoking status, systolic blood pressure (SBP), age and sex as covariates. The inclusion of these covariates is based on extensive literature of their potential influence on cerebral microstructure and composition including myelin content and axonal density. We note that the quadratic effect of age, age^2^, seen before (28) was not significant in most ROIs. This is likely due to the much-limited cohort size here and, therefore, the age^2^ term was omitted from the regression model. We also conducted a secondary analysis to investigate the association between PWV and AVF or MVF using the same regression model. In all cases, the threshold for statistical significance was *p* < 0.05. Due to the exploratory nature of this study and concerns about type 2 errors, multiple comparisons correction was not conducted. All calculations were performed within MATLAB (MathWorks, Natick, MA, United States).

## 3. Results

### 3.1. Participant demographic characteristics

Demographic characteristics of the participants are shown in Table 1. After excluding 16 participants with either cognitive impairment, missing PWV data, or bad quality images due to severe motion artifacts assessed using visual inspection, the final study cohort consisted of 38 cognitively unimpaired volunteers ranging in age from 22 to 94 years (mean ± standard deviation = 55.9 ± 21.2 years). Of this cohort, 20 (51.7%) were men and 18 (48.3%) were women, while 9 (23.7%) identified as smokers and 28 (73.7%) as nonsmokers. Among all 38 participants, only 2 patients were hypertensive with SBP values of 156 and 161 (mmHg). The mean ± standard deviation values of the PWV were 6.67 ± 1.04 (m/s), spanning a relatively large range between 5.1 and 11.2. All participants had a Mini Mental State Examination (MMSE) score higher or equal to 26 (29.1 ± 1.2).

**Table 1:** Demographic characteristics of participants of the study cohort.

Total sample	*N* = 38
Age (yrs.), mean ± SD (min–max)	55.9 ± 21.2 (22–94)
Sex	
Male, *N* (%)	20 (51.7%)
Female, *N* (%)	18 (48.3%)
Smoker	
Smokers, *N* (%)	9 (23.7%)
Nonsmokers, *N* (%)	28 (73.7%)
SBP (mmHg), mean ± SD (min–max)	116.6 ± 15.1 (95–161)
PWV (m/s), mean ± SD (min–max)	6.67 ± 1.04 (5.1–11.2)

SBP, systolic blood pressure; PWV, pulse wave velocity; SD, standard deviation; min, minimum; max, maximum.

**Figure 1:**
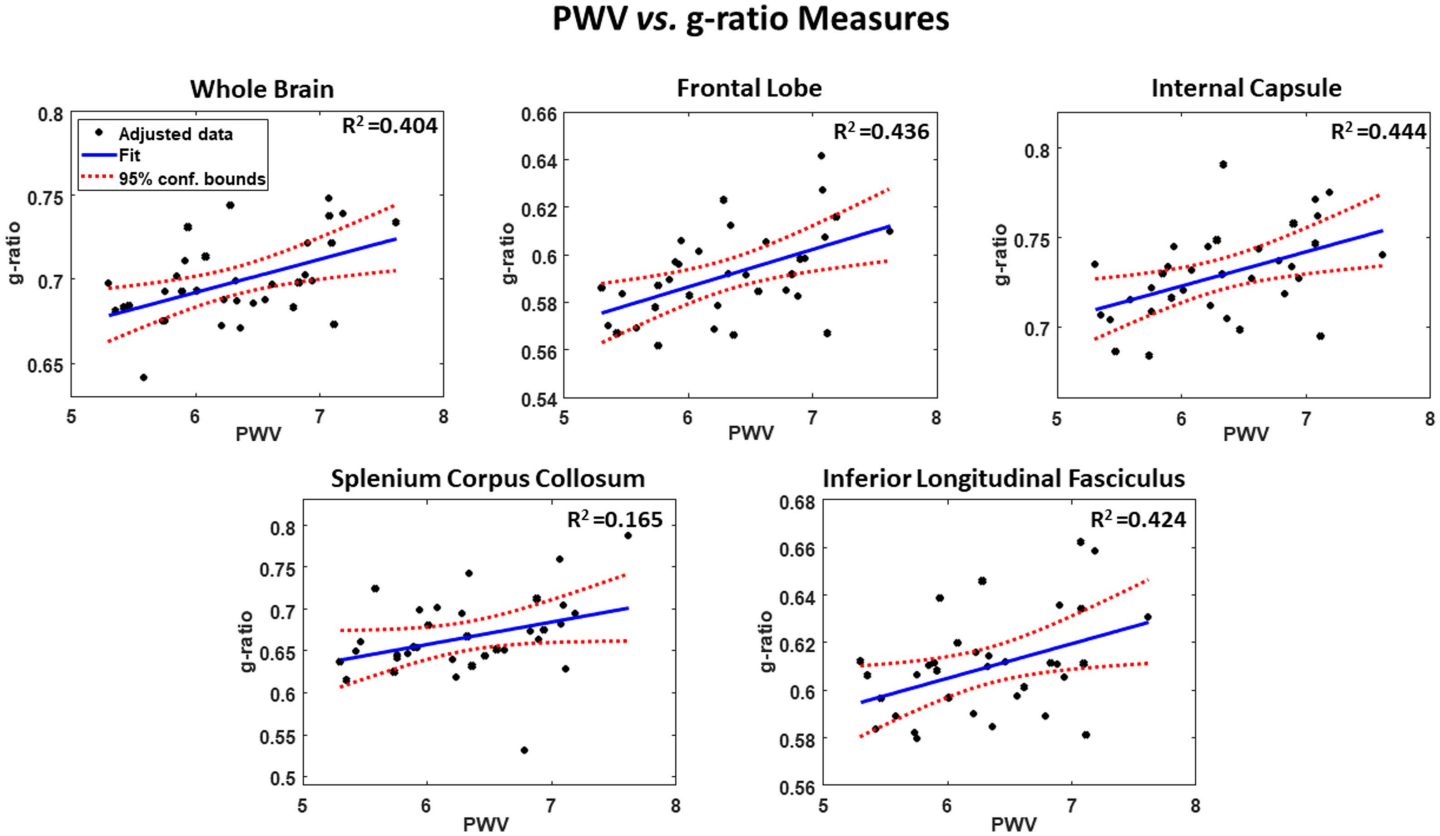
Example of regressions of *g*-ratio with pulse wave velocity (PWV), adjusted for age, sex, smoking, and SBP. Results are shown for 5 representative cerebral structures. For each plot, the coefficient of determination, *R*^2^, the line of best fit (blue solid line) and confidence intervals (red dashed lines) are displayed.

Figure 1 shows the results of the multiple regression analysis of aggregate *g*-ratio with PWV, after adjusting for age, sex, smoking status and blood pressure, for five representative cerebral structures. As seen, higher PWV values correspond to higher aggregate *g*-ratio values in all ROIs examined, with the best-fit curves displaying regional variation. Statistical analysis indicates that these positive correlations between aggregate *g*-ratio and PWV were statistically significant (*P*_PWV_ < 0.05) in several brain regions examined (Table 2), apart from the ROI defined by the occipital lobes. In addition, the greatest positive slopes in PWV with aggregate *g*-ratio were found in the splenium of the corpus callosum (SCC), the whole brain (WB), the internal capsules (IC), the corticospinal tract (CST), and the frontal and temporal lobes (FL, TL) regions, while the smallest slope was found in the occipital lobes. Furthermore, the effect of age was significant for several brain regions evaluated, especially in the white matter tracts (Table 2). As expected, besides the SCC, all ROIs exhibited positive slopes with age indicating higher aggregate *g*-ratio with age. Systolic blood pressure (SBP), a covariate in this experiment, showed statistical significance with aggregate *g*-ratio, with higher SBP values associated with lower aggregate *g*-ratio values in most of the brain regions analyzed. Finally, we note that the other covariates, namely, sex and smoking status, did not exhibit statistical significance in any of the investigated ROIs (data not shown), and virtually identical results, with very marginal differences in the regression coefficients and significances, and conclusions were obtained when excluding the data from the two hypertensive participants (data not shown).

**Table 2:** Regression coefficient (*β*) (standard error (*SE*) in italics), and significance (value of *p*) of *g*-ratio vs. PWV or age across 11 WM ROIs.

ROIs	*g*-ratio	
	PWV *β* (*SE*) × 10^−3^, value of *p*	Age *β* (*SE*) × 10^−4^, value of *p*
WB	19.9 (*6.61*), **0.006**	2.11 (*2.76*), 0.450
FL	15.9 (*5.13*), **0.004**	3.05 (*2.14*), 0.166
OL	4.55 (*6.73*), 0.505	3.53 (*2.81*), 0.219
PL	15.7 (*5.89*), **0.013**	2.25 (*2.46*), 0.368
TL	16.0 (*5.69*), **0.009**	4.35(*2.37*), 0.078
IC	19.3 (*6.86*), **0.009**	4.47 (*2.86*), 0.131
SCC	26.3 (*13.8*), 0.067	−8.29 (*5.75*), 0.161
ATR	15.2 (*5.82*), **0.015**	4.68 (*2.43*), 0.065
CST	17.8 (*5.57*), **0.004**	5.05 (*2.32*), **0.039**
IFOF	13.8 (*6.60*), **0.046**	5.07 (*2.75*), 0.076
ILF	14.5 (*6.04*), **0.024**	4.30 (*2.52*), 0.100

The multiple regression model is given by: *g* – ratio ~ *β*_0_ + *β*_age_ × age + *β*_PWV_ × PWV + *β*_Smoking_ × Smoking + *β*_sex_ × sex + *β*_SBP_ × SBP. The regression model accounted for sex and smoking status as categorical variables. Bolded values of *p* indicate statistical significance (*p* < 0.05).

AVF, axonal volume fraction; MVF, myelin volume fraction; PWV, pulse wave velocity; SBP, systolic blood pressure; WM, white matter; ROI, region-of-interest; WB, whole brain; FL, frontal lobe; OL, occipital lobe; PL, parietal lobe; TL, temporal lobe; IC, internal capsule; SCC, splenium corpus callosum; ATR, anterior thalamic radiation; CST, corticospinal tract; IFOF, inferior fronto-occipital fasciculus; ILF, inferior longitudinal fasciculus.

**Figure 2:**
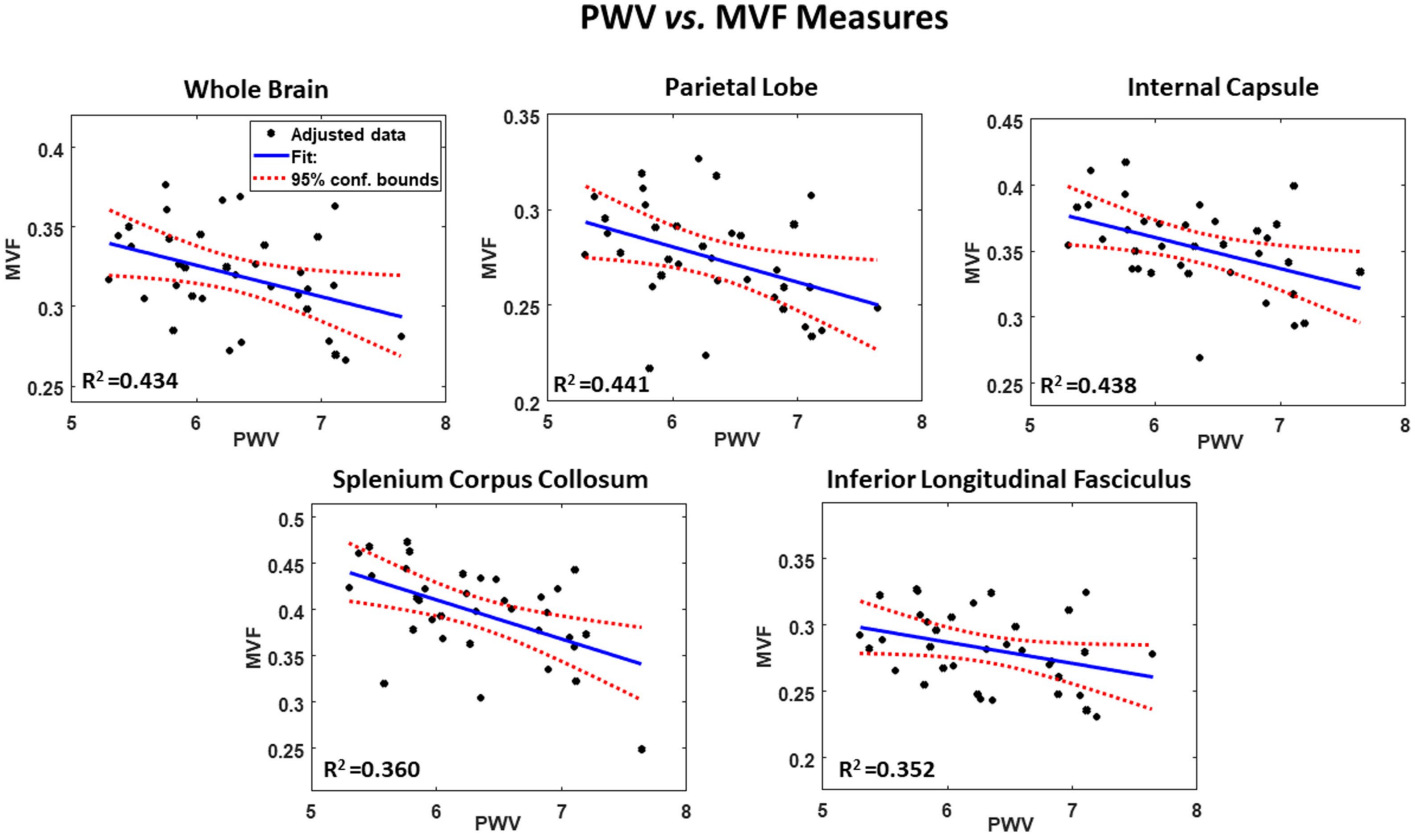
Example of regressions of MVF with carotid femoral pulse wave velocity (PWV), adjusted for age, sex, smoking, and SBP. Results are shown for 5 representative cerebral structures. For each plot, the coefficient of determination, *R*^2^, the line of best fit (blue solid line) and confidence intervals (red dashed lines) are displayed.

Our secondary analysis of the association between PWV and AVF or MVF showed that AVF exhibited non-significant associations in most ROIs examined (Table 3). However, the association between PWV and MVF was significant in most brain structures evaluated (Figure 2 and Table 3). Our statistical analysis indicated negative regional correlations between MVF and PWV, that is lower myelin content with higher arterial stiffness, that were statistically significant (*P*_PWV_ < 0.05) in several brain regions examined (Table 2), except for the ROIs defined by the frontal lobes, anterior thalamic radiation (ATR), and inferior fronto-occipital fasciculus (IFOF). Additionally, in overall agreement with the results of PWV vs. aggregate *g*-ratio (Table 2), the greatest negative slopes in PWV with MVF were found in the splenium corpus callosum (SCC), internal capsule (IC), and whole brain (WB). However, the smallest slope in PWV with MVF was found in the anterior thalamic radiation (ATR) (Table 3). Finally, while the effect of age was not significant, all ROIs, besides the SCC, exhibited negative slopes with age indicating lower MVF with age. SBP showed significant association with MVF, with higher SBP values associated with lower MVF values in most cerebral structures investigated. No significant association between SBP and AVF was identified. Here again, we note that the other covariates, namely, sex and smoking status, did not exhibit statistical significance with AVF or MVF in any of the investigated ROIs (data not shown).

**Table 3:** Regression coefficient (*β*) (standard error (*SE*) in italics), and significance (value of *p*) of AVF or MVF vs. PWV across 11 WM ROIs.

	AVF *β* (*SE*) × 10^−3^, value of *p*	MVF *β* (*SE*) × 10^−3^, value of *p*
WB	11.1 (*5.86*), 0.068	−19.5 (*8.55*), **0.030**
FL	10.2 (*3.82*), **0.013**	−15.0 (*9.28*), 0.118
OL	−0.54 (*3.68*), 0.884	−16.2 (*8.48*), 0.067
PL	6.55 (*4.46*), 0.153	−18.5 (*7.79*), **0.024**
TL	7.13 (*4.20*), 0.101	−15.0 (*7.48*), 0.055
IC	18.7 (*6.61*), **0.009**	−23.3 (*9.00*), **0.015**
SCC	13.3 (*10.8*), 0.226	−42.6 (*13.2*), **0.003**
ATR	4.98 (*5.05*), 0.333	−10.9 (*9.04*), 0.238
CST	9.99 (*5.90*), 0.102	−14.0 (*7.49*), 0.072
IFOF	4.88 (*4.93*), 0.331	−13.4 (*9.66*), 0.175
ILF	3.18 (*4.58*), 0.493	−16.0 (*8.12*), 0.059

The multiple regression model is given by: MRI ~ *β*_0_ + *β*_age_ × age + *β*_PWV_ × PWV + *β*_Smoking_ × Smoking + *β*_sex_ × sex + *β*_SBP_ × SBP, where MRI corresponds to axonal volume fraction (AVF) or myelin volume fraction (MVF). The regression model accounted for sex and smoking status as categorical variables. Bolded values of *p* indicate statistical significance (*p* < 0.05).

AVF, axonal volume fraction; MVF, myelin volume fraction; PWV, pulse wave velocity; SBP, systolic blood pressure; WM, white matter; ROI, region-of-interest; WB, whole brain; FL, frontal lobe; OL, occipital lobe; PL, parietal lobe; TL, temporal lobe; IC, internal capsule; SCC, splenium corpus callosum; ATR, anterior thalamic radiation; CST, corticospinal tract; IFOF, inferior fronto-occipital fasciculus; ILF, inferior longitudinal fasciculus.

## 4. Discussion

In this exploratory and cross-sectional study, we investigated the regional associations between aggregate *g*-ratio, derived from the BMC-mcDESPOT and NODDI MRI analyses (41, 42, 46), and pulse wave velocity (PWV), a measure of central arterial stiffness, in several cerebral white matter structures in a healthy cohort of adult participants spanning a wide age range. Our results showed regional correlations between aggregate *g*-ratio and PWV, with higher values of PWV associated with higher values in aggregate *g*-ratio, indicating lower microstructural integrity. These associations were statistically significant in several white matter regions investigated, especially in the regions that have been widely documented to be sensitive to arterial stiffness—namely, the splenium of the corpus callosum, the internal capsules and the white matter tracts (14–16, 22–24). Our results provide further evidence of the intimate relationship between vascular physiology and cerebral white matter integrity.

Our results agree with previous MRI investigations indicating that lower microstructural integrity of cerebral white matter is associated with higher PWV, in both young and older adults (14–16, 22–24). Using diffusion tensor imaging (DTI), an advanced quantitative MRI technique to probe cerebral microstructure, previous investigations have consistently shown that increased PWV is associated with lower FA and higher RD values, while exhibiting weak to no association with AxD; these DTI metrics are very sensitive to differences in cerebral tissue integrity (18, 19). According to these results, increased deterioration in myelin integrity is associated with increased arterial stiffness. However, while it is commonly assumed that FA reflects myelin and axonal integrity, and that RD and AxD are, respectively, sensitive metrics of axonal demyelination and axonal loss, these DTI metrics are not specific due to their sensitivity to several physiological and experimental confounding factors. Therefore, using our advanced MR methodology with multicomponent relaxometry and diffusion (28), and combining our measures of MVF and AVF to quantify myelin content and axonal density, respectively, we were able to more thoroughly explore the association between arterial stiffness and myelin and axonal integrity. To the best of our knowledge, our study is the first to investigate this relationship using these specific measures. Interestingly, and in agreement with the previous DTI investigations, our secondary analysis indicates that the relationship between PWV and aggregate *g*-ratio was mostly driven by differences in myelin content rather than axonal density. Indeed, our results showed significant regional associations between higher PWV and lower MVF in almost all ROIs investigated, while this association with AVF was not significant in the majority of ROIs. The sensitivity of oligodendrocytes, the main cells responsible for synthesizing and maintaining myelin, to arterial stiffness is expected. In fact, it has recently been shown that deficits in cerebral blood flow are associated with a lower regional myelin content, including in cognitively unimpaired adults (11–13). Therefore, any change in blood supply to the brain, due for example to a decreased elasticity of the arteries and subsequent impaired baroreflex, is expected to affect cerebral tissue integrity, especially in the myelination process. Such changes in biomechanical characteristics of the vasculature could result in vascular alterations and hemodynamic stress which may ultimately limit the delivery of oxygen, glucose and essential nutrients to oligodendrocytes.

The well-documented regions of the splenium corpus callosum (SCC) and the internal capsules (IC), which are sensitive to arterial stiffness, exhibited the highest slopes in the relationships between PWV and aggregate *g*-ratio or MVF. These results support previous MRI findings indicating greater deterioration in the microstructure of these cerebral regions with increased arterial stiffness (14–16, 22–24). These brain structures as well as the white matter tracts, which also exhibit significant associations between aggregate *g*-ratio or MVF and PWV, are supplied by the anterior and middle cerebral arteries making them particularly sensitive to arterial stiffness. The microstructure of these regions has been shown to be compromised in various neurodegenerative diseases, especially Alzheimer’s and dementias, making them potential biomarkers of arterial stiffening in dementias’ pathophysiology. Moreover, we also identified additional cerebral regions that exhibited significant associations between aggregate *g*-ratio or MVF and PWV, namely the frontal, parietal and temporal lobes. Interestingly, the occipital lobes did not exhibit significant associations between aggregate *g*-ratio or MVF and PWV. The relative sensitivity of the anterior brain regions as compared to the posterior regions seems to adhere to the retrogenesis paradigm, in which posterior brain regions are spared from degeneration as compared to anterior brain regions (48–50). However, further analyses on larger cohorts are needed to confirm this observation.

The cross-sectional design of this study limits our ability to establish a causal relationship between arterial stiffness and aggregate *g*-ratio in aging adults. In addition, the small sample size may lead to overfitting issues. Further longitudinal studies using larger datasets are needed to confirm the trends observed in this work. Additionally, partial volume effects, as well as contamination from cerebrospinal fluid in areas with limited spatial resolution, could have been introduced. Although these issues were analyzed prior to data analysis, some partial volume effects could have persisted, introducing some bias into derived aggregate *g*-ratio values. Moreover, as with all quantitative MRI methods, physiological and experimental factors that are not taken into consideration as well as underlying model assumptions could bias determination of derived parameters. These include, but are not limited to, magnetization transfer between macromolecules and free water protons, *J*-coupling, spin locking, iron content, exchange between water pools, off-resonance effects, water diffusion within different compartments, and internal gradients. Therefore, derived MVF and AVF values are dependent on these parameters so that additional studies using other MRI techniques to measure aggregate g-ratio are still required (29, 30, 51–54). Finally, we note that while the regression analysis adjusted for the blood pressure, the later may have varied between the time of its measure and the time of the MRI. Nevertheless, since all measures were conducted within a few hours, no major difference would be expected.

## 5. Conclusion

Our study shows that aggregate *g*-ratio is positively correlated to arterial stiffness, in several white matter structures in the adult human brain free of cognitive impairments. These regional associations appeared to be mostly driven by differences in myelin content.

## Data availability statement

The raw data supporting the conclusions of this article will be made available by the authors, without undue reservation.

## Ethics statement

The studies involving human participants were reviewed and approved by the MedStar and National Institute on Aging IRB. The patients/participants provided their written informed consent to participate in this study.

## Author contributions

MB: experimental design. JL, MF, ZG, EP, MA, and MB: analysis and paper editing. MB and JP: paper writing. All authors contributed to the article and approved the submitted version.

## Funding

This work was supported by the Intramural Research Program of the National Institute on Aging of the National Institutes of Health.

## Conflict of interest

The authors declare that the research was conducted in the absence of any commercial or financial relationships that could be construed as a potential conflict of interest.

## Publisher’s note

All claims expressed in this article are solely those of the authors and do not necessarily represent those of their affiliated organizations, or those of the publisher, the editors and the reviewers. Any product that may be evaluated in this article, or claim that may be made by its manufacturer, is not guaranteed or endorsed by the publisher.
